# Focused multidimensional scaling: interactive visualization for exploration of high-dimensional data

**DOI:** 10.1186/s12859-019-2780-y

**Published:** 2019-05-02

**Authors:** Lea M. Urpa, Simon Anders

**Affiliations:** 1 Institute for Molecular Medicine Finland (FIMM), University of Helsinki, Helsinki, Finland; 2 Center for Molecular Biology of the University of Heidelberg (ZMBH), Heidelberg, Germany

**Keywords:** Clustering, High-dimensional data, Visualization, Personalized medicine

## Abstract

**Background:**

Visualization is an important tool for generating meaning from scientific data, but the visualization of structures in high-dimensional data (such as from high-throughput assays) presents unique challenges. Dimension reduction methods are key in solving this challenge, but these methods can be misleading- especially when apparent clustering in the dimension-reducing representation is used as the basis for reasoning about relationships within the data.

**Results:**

We present two interactive visualization tools, distnet and focusedMDS, that help in assessing the validity of a dimension-reducing plot and in interactively exploring relationships between objects in the data. The distnet tool is used to examine discrepancies between the placement of points in a two dimensional visualization and the points’ actual similarities in feature space. The focusedMDS tool is an intuitive, interactive multidimensional scaling tool that is useful for exploring the relationships of one particular data point to the others, that might be useful in a personalized medicine framework.

**Conclusions:**

We introduce here two freely available tools for visually exploring and verifying the validity of dimension-reducing visualizations and biological information gained from these. The use of such tools can confirm that conclusions drawn from dimension-reducing visualizations are not simply artifacts of the visualization method, but are real biological insights.

**Electronic supplementary material:**

The online version of this article (10.1186/s12859-019-2780-y) contains supplementary material, which is available to authorized users.

## Background

Visualization is key for understanding patterns and generating meaning from scientific data. High-dimensional data, however, presents unique challenges in that patterns or structures may exist only in greater than three dimensions, and these relationships often cannot be visualized exactly in two- or three-dimensional space. One example is the analysis of data from comparative high-throughput sequencing experiments, where a key quality-assessment step is to explore the similarity between samples in order to see whether the replicate samples are similar and to spot outliers. Samples are plotted as points on a two-dimensional (2D) plane, such that the relative position of points to each other represent the relationships between the samples. Popular ways to create this kind of visualization include principal components analysis (PCA), which plots the components of the data that explain the most variability, or multidimensional scaling (MDS), which attempts to capture the relationship between the points across all measures and represent it in 2D space.

Similarly, in single-cell RNA sequencing (RNA-seq) one often wishes to reduce high-dimensional expression data to a 2D plot, such that cells with similar transcriptomes appear close together. Here, besides PCA and MDS, t-distributed stochastic neighbor embedding (t-SNE) [1] and uniform manifold approximation and projection (UMAP) [2] have become methods of choice. t-SNE is an optimization algorithm that uses probability distributions in high and low dimensional space to generate 2D or 3D representations, while UMAP is a manifold learning technique based in Riemannian geometry and algebraic topology. A third illustrative example that we will use in this paper are experiments which investigate the effect of a panel of drugs on a collection of cancer patient biopsies, with one objective being the identification of groups of patient samples with similar sensitivities to drugs, e.g. Heckman et al. [3]. We can easily pick any two patient samples and compare, say, the correlation coefficient between their respective sensitivities to the panel of drugs, but providing a visual overview of the similarities between all the patient samples requires some means of dimension-reducing visualization.

**Fig. 1:**
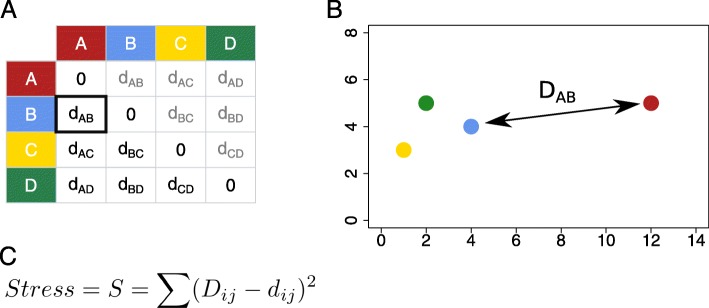
Schematic representation of the strategy for multidimensional scaling. **a** An example positive, symmetric matrix of distance values between four objects. **b** A dimension-reducing MDS representation of the distances in the matrix. **c** The stress equation for calculating the overall difference between the distances in the feature space (panel A, *d*_*i*,*j*_) and the distances on the 2D plane (panel B, *D*_*i*,*j*_)

In each of these examples the aim of the dimension reduction is the same: to arrange the points representing individuals (samples, cells, or drugs) on a two-dimensional plot such that the closeness between points on the plot represents as well as possible the objects’ similarities. While PCA is commonly the first method that comes to mind to create such a plot, MDS is arguably closer to the goal of representing the objects’ overall similarity to one another. MDS takes as an input a symmetric matrix with distances, or “dissimilarity scores”, for all pairs of samples. From these distances, the algorithm numerically searches for a placement of points on the plot that minimizes “stress” (Fig. 1c), the discrepancy between the actual or “feature space” distances and the distances of the points embedded in the 2D plane, summed over all pairs of points (see Fig. 1). No arrangement can exactly represent the distances between all points in all dimensions, unless the data was already in a two-dimensional sub-space to start with, and hence any MDS (or other dimension-reducing representation) must make some trade-offs in accurately depicting the relationships between objects. All dimension-reducing visualizations are therefore bound to be misleading with respect to at least some of the objects depicted, and might even be misleading for a substantial part of them. This issue of misleading depiction is particularly important when dimension-reducing visualizations are suggestive of clusters or other structures in the data. As is often emphasized in the field of single-cell RNA sequencing, formally inferring clusters or other structures should be done on the full feature space data rather than on the dimension-reduced embedding. Nevertheless, dimensional reduction is meant to give the viewer an intuitive grasp of the data, and therefore it is important to be able to determine the validity of any structure that one might see in such a visualization. Such validation is possible via statistical means [4–9], but the tools for exploring the validity of dimension-reduction visualizations visually are limited.

**Fig. 2:**
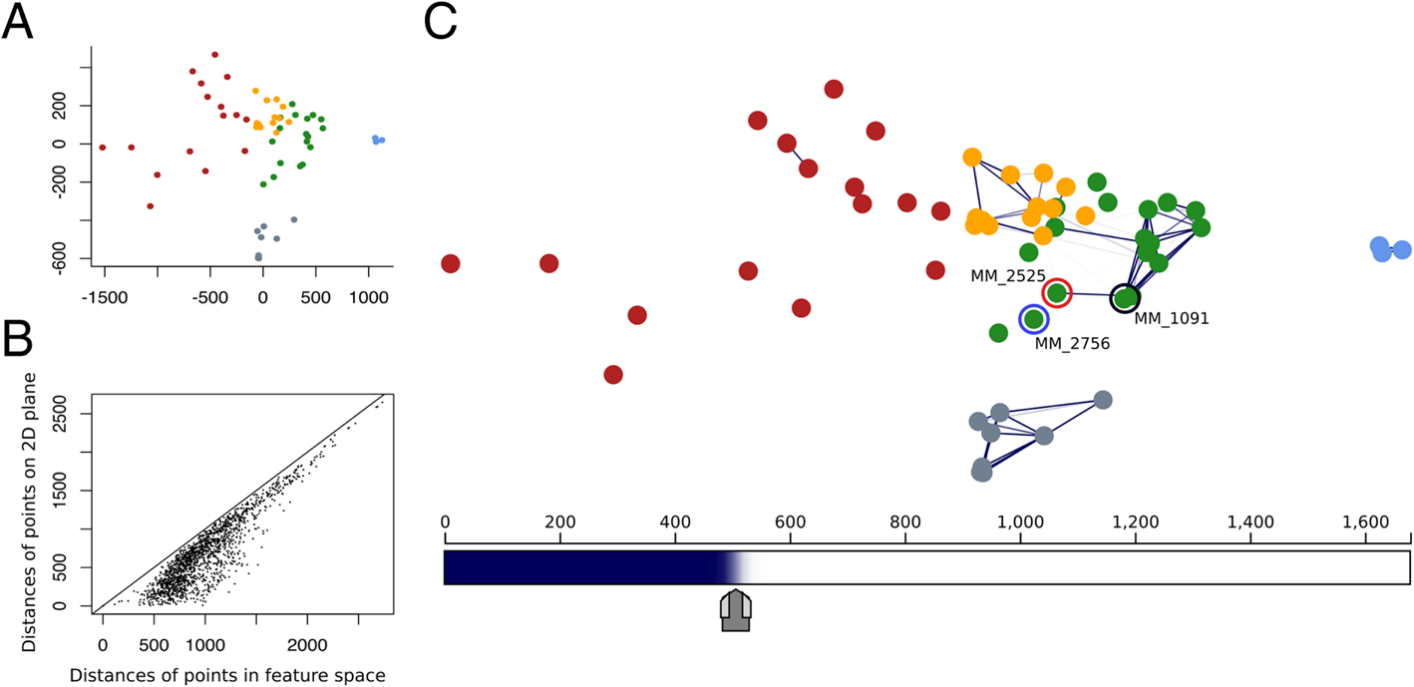
Dimension reduction and *distnet* representation of differences in *ex vivo* drug sensitivities between hematological cancer patient samples from Majumder et al. [10]. **a** A standard multidimensional scaling representation of the differences in drug sensitivity between patient samples. **b** The distances between points in panel A compared to their actual distances in the feature space (a Shepard plot). **c** A static version of the *distnet* plot of this dataset, where the lines between points represent point pairs with a distance of 500 or less in the feature space. Circled points show inconsistencies between the feature space distances and distances on the 2D plot. For an interactive version of panel C, visit the supplemental interactive file online [15]

We illustrate this using data from Majumder et al. [10], who tested a panel of 308 drugs ex vivo on 58 samples from hematological cancer patients and identified four stratified patient groups. Each patient sample is described by a vector comprised of the sensitivity score measured for each of the 308 drugs (see Methods for details on how these scores are calculated). One may expect that the response profiles are similar for patients whose cancers have similar molecular characteristics, and hence expect to see them clustering together in a dimension-reducing visualization. We therefore calculated Manhattan distances between the vectors of drug sensitivity scores for each sample and visualized them in the MDS plot in Fig. 2a, using the isoMDS function from R’s MASS package [11], a commonly used MDS function in R. Colors indicate the stratified patient groups as defined by Majumder et al. [10] via hierarchical clustering on Manhattan distances. Figure 2b plots the distance between all pairs of samples in the MDS plot against their actual feature space distance. This so-called Shepard plot shows that the agreement between the feature space distance and the distance on the 2D plane is quite unsatisfactory: many points with small distances on the 2D plot have quite large actual feature space distances, suggesting that the plot might not be suitable for assessing the validity of the patient groupings.

Here we present two interactive visualization tools, called *distnet* and *focusedMDS*, which offer ways to explore multidimensional data in a manner that safeguards against misleading depiction. The *distnet* tool uses a distance net visualization to explore the validity of existing dimension reduction plots, while *focusedMDS* provides an alternative method of multidimensional scaling that gives a true picture of one “focal” point in relation to all others. These tools are designed to visually explore multidimensional data, complementing existing exploratory data visualization methods such as correlation heatmaps and dendrograms.

## Implementation

Both tools are provided as R packages, and can be installed with the R commands install.packages(“focusedMDS”) and devtools::install_github(“simon-anders/distnet”). As documentation, an interactive introduction for both packages is available online [12]. The most recent unreleased development versions are available on GitHub [13, 14].

## Results and discussion

### *distnet*

The *distnet* tool takes a data frame of 2D coordinates from a dimensional reduction method and a corresponding distance matrix (as produced by R’s *dist* function, for example). The dimension reduction visualization is then reproduced (Fig. 2c) with the addition of a scale bar and color bar at the bottom of the plot. This scale bar shows the minimum and maximum pairwise distances between the pairs of points in the original feature space, with all pairwise distances in the data in between. The slider may be moved back and forth along the color bar, and movement of the slider will connect on the plot any pair of points with pairwise distances less than or equal to the slider’s location on the scale. This threshold is represented by a gradient of colors, where dark blue is used for distances well below the threshold and distances near the threshold gradually fade to white. The threshold can also be “softened” or “hardened” by dragging the wings of the slider, widening or narrowing the range of the gradient. If no 2D coordinates are provided, the points are placed according to a Kruskal MDS dimensional reduction, calculated using isoMDS [11]. Text labels and colors for the points may also be provided.

Figure 2c shows the data from Majumder et al. [10] as depicted in *distnet*. The coordinates from the MDS plot shown in Fig. 2a were input to *distnet*, which displays the dimension reduction visualization and the additional scale bar and color bar. This allows us to spot some explicit discrepancies in the MDS plot of the data. For example, judging only from the distances of the points on the plot, the *ex vivo* sample from patient MM_2525 (outlined in red) looks more similar to the sample from patient MM_2756 (outlined in blue), while in fact the sample’s drug profile is actually much closer to MM_1091 (outlined in black). A line connects sample MM_2525 to MM_1091, indicating that the pairwise distance between the two is at least 500, and the lack of a line between MM_2525 and MM_2756 indicates their pairwise distance must be greater than 500. Therefore, despite the closeness of samples MM_2525 and MM_2756 on the plot, the drug profile for sample MM_2525 is actually closer to sample MM_1091. This is one example– this paper’s HTML supplement (available as Additional file 1 and online [15]) provides an interactive version of this figure, where the user can vary the threshold to interactively explore the similarity relationships of the samples and search for more inconsistencies. The interactive version of Fig. 2c in the supplement can be viewed in any web browser with Javascript enabled.

This kind of interactive plot is a useful way to explore the validity of a dimension-reducing visualization of distance data, be it from MDS, PCA, t-SNE, UMAP, or any other similar method. This is important, as it has become quite common to reason about relationships between entities based only on a dimension-reducing visualization. In single-cell RNAseq profiling, for example, t-SNE plots are often used directly to infer biological insights such as the existence of cellular subtypes. Again, formally inferring clusters or other structures in the data should be done using the full feature space data, not the lower-dimension embedding. Yet the prevalence of using such dimension-reducing visualizations to reason about the relationships between objects shows that visualization is a powerful tool in understanding data, even if it can be misleading. Previously, only indirect ways to explore the validity of such visualizations has been possible: through validating the identified clusters via statistical methods [4–9]. While these methods are important and useful, they do not help in identifying and understanding why the reasoning about relationships in the data based on a dimension-reducing visualization are incorrect. The *distnet* tool is a complementary method that provides a visual means to directly explore the validity of clusters or other apparent structures in a dimension-reducing visualization.

### *focusedMDS*

Figure 2c shows that for the data from Majumder et al., MDS might not be the best dimension reduction tool to visualize the similarities and differences in drug response between patient samples, and that it would be misleading to directly infer drug response groups from such a visualization. In fact, the authors stratified the patient samples into response groups based on unsupervised hierarchical clustering of the drug sensitivity data, not based on such a dimension-reducing visualization. We have then answered the question of whether the MDS plot from Fig. 2 was a good representation of the relationships in the data, but we have not actually explored whether the patient response groups as classified by Majumder et al. via hierarchical clustering are meaningful. A dimension-reducing visualization would be a useful tool in exploring these groups classifications, but it seems that standard MDS is not a good choice here. When considering another dimension reduction algorithm, we must bear in mind that all dimension-reducing plots must make some trade-offs, as no algorithm can exactly represent the relationships between all objects in all dimensions. In the context of personalized medicine, we want to focus on a single patient that may need to be treated differently than others, even within its stratified group. We can then decide that it is useful to very accurately depict the relationship of one sample in particular to all others, even if it is at the expense of accurately depicting the relationships between the samples we are not focusing on. To this end, we have created a visualization tool that shows the distances of one “focal point” to all others exactly, while depicting the distances between the rest of the points as accurately as possible.

**Fig. 3:**
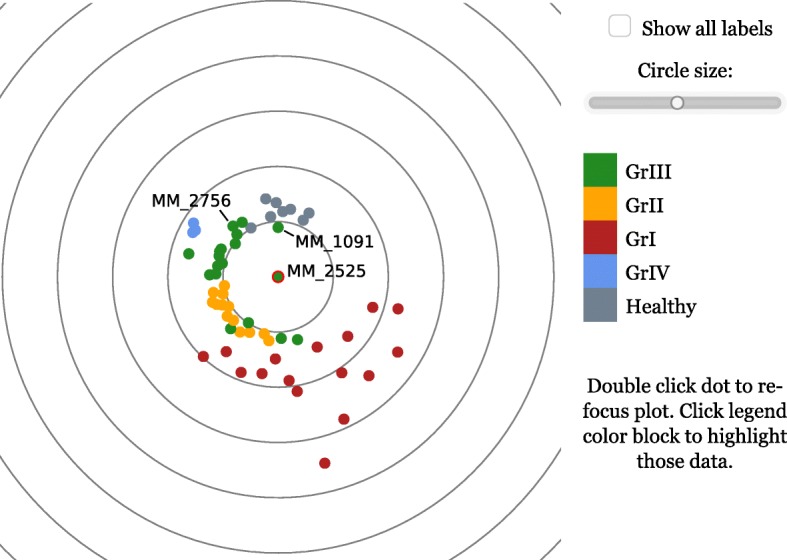
*focusedMDS* representation of drug sensitivity score distance data. The drug sensitivity score data from [10] in Fig. 2, visualized with *focusedMDS*. The three samples flagged in Fig. 2 are again identified. This is a static picture of the focusedMDS app– for an interactive version, visit the online manuscript supplement [15]

The focusedMDS tool takes a distance matrix containing pairwise dissimilarity measures between points (either produced by R’s dist function, or simply any symmetric, positive matrix with zero diagonal that fulfills the triangle inequality). The function creates an interactive plot (Fig. 3), where one “focal point” is plotted at the center of the figure, and all other points are plotted around this point. We can imagine that a non-focal point is placed on a circle around the focal point, where the radius of that circle is the exact distance of the point *i* to the focal point. The angle *ϕ*_*i*_ at which the point is placed on its circle of radius *r*_*i*_ is determined by the relationship of the point to the rest of the non-focal points. We choose a *ϕ*_*i*_ for the point that minimizes *stress*, the difference between the distance of point *i* to the rest of the non-focal points on the 2D plot and the distances in the feature space (see the Methods for a mathematical description of this method). Therefore the distances between the focal point and all other points are shown exactly, via the fixed *r*_*i*_ of the polar coordinate, while the relationships between the non-focal points are depicted as accurately as possible, by minimizing stress when choosing the *ϕ*_*i*_ coordinate for each point. Double clicking on any point will move that point to the center of the plot, and all other points will be arranged around this new focal point such that the distances to the new point are now represented exactly.

**Fig. 4:**
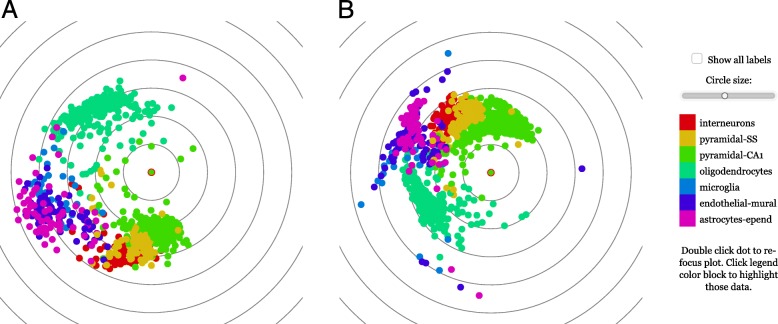
*focusedMDS* representation of single cell mouse brain transcript data. Individual mouse brain cells forming cell type-specific clusters based on single-cell gene expression information, data from Zeisel et al. [16], with focusedMDS generated from correlation distances (panel **a**) and Euclidean distances (panel **b**). A cluster of neuronal cells (interneurons as red, pyramidal somatosensory cortex (SS) as yellow and hippocampal pyramidal CA1 neurons as green) can be seen to form a separate cluster from oligodendrocytes (lime) and support cells (microglia as blue, endothelial-mural cells navy, and astrocyte-ependymal cells as purple), though some cells appear to be between the defined clusters. To interactively explore this dataset, visit the online manuscript supplement [15]

Circular lines are added in the background of the plot to help judge distances between the focal point and other points. Hovering over any point will reveal the text label of the point; if no text labels are given, a number will be assigned. If group assignments for the points are given, a legend appears with names of the groups and colors. Hovering over the group color in the legend will highlight only that group, and clicking on one or more legend colors will highlight multiple groups. The size of the points in the plot can also be adjusted with a slider. The focusedMDS app works well with up to 1000 points; beyond this, limitations of browser capabilities may restrict the functionality of the plot or make rendering too slow. Figures 3 and 4 show static examples of the focusedMDS tool, but the HTML supplement [15] provides live, interactive versions of these figures.

Figure 3 shows a static version of the focusedMDS plot created from the same Majumder et al. [10] data as from Fig. 2. The data was classified into patient response groups based on unsupervised hierarchical clustering of the distances between patient drug sensitivity scores, which uses a variable threshold to determine the number of clusters and cluster identity. While we do not dispute the validity of the clusters identified in the paper, with this method all samples are classified into groups, even if some may not be typical representatives of any group (and some groups may be more meaningful than others). In a personalized medicine context, it may be worthwhile to ask whether a particular patient sample is a typical representative of a group, or a marginal case. In Fig. 3, we can see that the focal point (MM_2525) assigned to group three (GrIII) is as close to the other green points of GrIII as it is to the yellow and grey points of the group two (GrII) and Healthy groups. In this case, sample MM_2525 appears to be a marginal case, rather than a typical representative of the group. Because the patient sample strata may be used for treatment recommendations, it may be the case that marginal patient samples such as MM_2525 should be treated differently than typical representatives of the group when giving such advice. This closeness of this sample to the two different groups is not immediately apparent in the dendrogram visualization of the original manuscript. This does not mean that the patient stratification described by the authors is incorrect or not useful- stratification of patients with refractory multiple myeloma into treatment groups via *ex vivo* drug testing is a significant advancement in personalized medicine for patients whose options are otherwise limited. But by visualizing individual patients in the stratified group in this focused manner, researchers and clinicians can understand whether a particular case is a good representative of the patient strata, or if further investigation into the drug sensitivity data is warranted.

The *focusedMDS* tool is also useful in contexts other than personalized medicine, particularly when exploring group classifications within data. As an example from a different field, Fig. 4 plots individual mouse brain cells from Zeisel et al. [16], where distances between cells are calculated based on single-cell RNA expression (correlation distances in panel A, and Euclidan distances in panel B; see Methods for details). This visualization shows clusters of neurons (interneurons, pyramidal somatosensory cortex and pyramidal hippocampus CA1 neurons) as distinct from clusters of oligodendrocytes and support cell populations (microglia, endothilial-mural, and astrocyte-ependymal cells). The plot reiterates the finding from Zeisel and colleagues that single-cell RNA-seq can effectively distinguish between neuronal and other cell types, but when exploring this data with focusedMDS the user can see that there are a substantial number of cells whose identity is somewhere between the identified clusters. Again, an interactive version of this figure is available in the HTML supplement [15]. One can hence see the usefulness of focusedMDS for exploring or verifying how robust cluster assignments are.

## Conclusions

The distnet and focusedMDS packages are useful tools for exploring multidimensional data, both by investigating the relationship between a dimension-reducing visualization and its underlying multidimensional data, and by visualizing such data in a novel way. While no two-dimensional representation of high dimensional data can completely represent the relationships in the data, the *distnet* tool is particularly useful for investigating existing dimension reduction visualizations and the biological insights gained directly from these, while *focusedMDS* is most useful when exploring the relationship of one particular individual to the rest of the samples. The use of these tools can increase confidence that conclusions drawn from dimension-reducing visualizations are not simply artifacts of the visualization method, but are real biological insights.

## Methods

### Computational methods

The *distnet* and *focusedMDS* tools are implemented in Javascript using M. Bostock’s D3 library [17], a framework for developing interactive data visualization with Javascript. For univariate minimization, we manually translated the Fortran code of *fmin* in the NetLib FMM library [18] to JavaScript. The *htmlwidgets* package [19] was used to construct R wrappers around the Javascript code, making the tools available as R packages.

### *focusedMDS* mathematical method

The *focusedMDS* tool visualizes distance matrix information, given a matrix of values *d*_*ij*_ indicating feature space distances between all pairs of points *i* and *j* (where *d*_*ij*_=*d*_*ji*_ and *d*_*ii*_=0). Points are added iteratively in polar coordinates from the focus point outward. For each new point, the radius *r*_*i*_ is given by the distance to the focus point (*d*_1,*i*_). The angular coordinate *ϕ*_*i*_ of the new point is chosen to minimize the stress, ${\sum \nolimits }_{j} S_{ij}$, between previously placed points *j* and the new point *i*, where *S*_*ij*_ is given by (*D*_*ij*_−*d*_*ij*_)^2^, i.e. the squared difference between the points’ given feature space distance *d*_*ij*_ and the distance of their representatives (*r*_*i*_,*ϕ*_*i*_) and (*r*_*j*_,*ϕ*_*j*_) on the 2D plot, called *D*_*ij*_ (see Fig. 1). The minimizing *ϕ*_*i*_ is found using the univariate numerical optimization algorithm of Brent [20]. By using iterative univariate optimization, we avoid the computationally costly multivariate optimization strategy of minimizing stress between all points at once. This allows for fast, interactive visualization of the high-dimensional data in an intuitive way.

### Example data methods

For Figs. 2 and 3, data from Majumder et al. [10] were obtained from the authors. We calculated Manhattan distances between the 58 multiple myeloma patient samples based on their *ex vivo* drug sensitivity scores (DSS) for 308 clinical and emerging oncology drugs. Drug sensitivity score, as described in Majumder et al. [10], is an area-under-the-curve-like sensitivity score calculated from dose-response cell viability measurements at five drug concentrations for each drug. Simple Manhattan distances between the vectors of DSS values were calculated using the dist function from the R base statistical methods [21], and the assignment of patients to groups are those published in Majumder et al. [10].

For Fig. 4, we obtained gene expression data for individual mouse brain cells from Zeisel et al. [16], Fig. 1, by communication with the authors. We performed quality control on the gene counts as described in the supplementary methods of Zeisel et al.. Briefly, we removed any cells with less than 2500 total RNA molecules detected and any genes with less than 25 molecules detected over all cells. We then calculated a correlation matrix over all genes, defined a threshold as the 90th percentile of this matrix (0.2064), and removed any genes which had less than 5 other genes that correlated more than this threshold.

For the subsequent processing, we followed a standard workflow that is also used by the Seurat package [22] for single-cell transcriptomics data analysis: we normalized the unique molecular identifier (UMI) counts given in the expression matrix by dividing, for each cell, the count for each gene by the total count for that cell. We then multiplied each normalized count by 10^3^, added a pseudocount of 1, and performed a log2 transformation. For Fig. 4a, we then chose the top 200 most variable genes and calculated 1 minus the Spearman correlation between those genes. For Fig. 4b, again following the Seurat package’s [22] standard workflow, we calculated the first 50 principal components of the normalized, log-transformed counts and used these components to calculate Euclidean distances with R’s *dist* function [21].

## Availability and Requirements

**Project name:** focusedMDS, distnet

**Project home page:**https://github.com/anders-biostat/focusedMDS and https://github.com/simon-anders/distnet/

**Operating system(s)**: Platform independent

**Programming language:** R, Javascript

**Other requirements:** R version greater than 3.3.1, R packages htmlwidgets (0.6 or higher), MASS, grDevices

**License:** GNU General Public License

**Any restrictions to use by non-academics:** none

## Additional file

Additional file 1 HTML file corresponding to https://lea-urpa.github.io/PaperSupplement.html. To view the file, download the zip file, unzip, and double click the HTML file to open in any browser with Javascript enabled. (ZIP 2891 kb)

## Abbreviations

2D, 3D: Two dimensional, three dimensional
DSS: Drug sensitivity scores
focusedMDS: Focused multidimensional scaling
GrII, GrIII: Group two, group three
MDS: Multidimensional scaling
PCA: Principal components analysis
RNA-seq: RNA sequencing
t-SNE: T-distributed stochastic neighbor embedding
UMAP: Uniform manifold approximation and projection
UMI: Unique molecular identifier
