# Supplementary material for: Focused multidimensional scaling: interactive visualization for exploration of high-dimensional data
Source: BMC Bioinformatics. 2019 May 2;20:221. doi: 10.1186/s12859-019-2780-y (PMC6498510; doi:10.1186/s12859-019-2780-y)
Supplement: Supplementary file 1 — HTML file corresponding to https://lea-urpa.github.io/PaperSupplement.html. To view the file, download the zip file, unzip, and double click the HTML file to open in any browser with Javascript enabled. (ZIP 2891 kb) [file 12859_2019_2780_MOESM1_ESM.zip › urpa_focusedMDS_supplement/PaperSupplement.html]

- Homepage
- focusedMDS
- Paper Supplement
- About Author

# Paper Supplement

HTML supplement to the `focusedMDS` manuscript submission.

## Figure 2

Supplement to Figure 2 of the focusedMDS manuscript submission, showing visualization of data from
 Majumder *et al.* 2017  with  distnet .

## Figure 3

Supplement to Figure 3 of the focusedMDS manuscript submission, showing visualization of data from
 Majumder *et al.* 2017  with focusedMDS.

## Figure 4

Supplement to Figure 4 of the focusedMDS manuscript submission, showing visualization of data from
 Zeisel *et al.* 2015 
with focusedMDS.

- Linked In
- Linked In
- Twitter

Design by TEMPLATED.
